# Co-occurrence of *mcr-9* and *bla*_NDM-1_ in carbapenem-resistant *Enterobacter hormaechei* from burn patients

**DOI:** 10.3389/fcimb.2026.1742596

**Published:** 2026-02-13

**Authors:** Xingchen Tao, Lingyi Zeng, Xinyi Sun, Yuhai Du, Liping Zhong

**Affiliations:** 1 Department of Laboratory, Jiaxing Maternal and Child Health Care Hospital (Affiliated Women’s and Children’s Hospital of Jiaxing University), Jiaxing, China; 2 Microbiology Laboratory, Hangzhou Center for Disease Control and Prevention (Hangzhou Health Supervision Institution), Hangzhou, China

**Keywords:** *bla*_NDM-1_, carbapenem-resistant *Enterobacter hormaechei*, colistin, *mcr-9*, whole-genome sequencing

## Abstract

**Background:**

Carbapenem-resistant *Enterobacter* (CRE) has emerged as a critical clinical concern due to its broad multidrug resistance. This study aims to characterize the features of clinical CRE strains co-harboring the *bla*_NDM-1_ and *mcr-9* genes from three burn patients.

**Methods:**

This study collected 110 non-repetitive carbapenem-resistant *Enterobacteriaceae* (CRE) from clinical settings. *bla*_NDM-1_ and *mcr-9* genes were identified by PCR, and strains were identified via MALDI-TOF MS and 16S rRNA sequencing. Minimum inhibitory concentrations (MICs) of common antimicrobial agents were determined by the broth microdilution method. The conjugation experiment was used to verify the transfer of resistance plasmids. Whole-genome sequencing (WGS) was performed using NovaSeq and PacBio_HIFI platforms, and analyzed through bioinformatics to characterize the resistance genes, virulence factors, plasmid profiles, and genetic relatedness of the bacterial strains.

**Results:**

Three carbapenem-resistant *Enterobacter hormaechei* (CR-*E. hormaechei*) strains co-harboring *bla*_NDM-1_ and *mcr-9* were isolated from burn patients, and exhibited broad multidrug resistance with 100% conjugation efficiency. Whole-genome sequencing (WGS) showed that CR-1025 and CR-1050 carried 12 additional resistance genes (targeting aminoglycosides, β-lactams, etc.), while CR-1051 harbored 9. The *mcr-9* gene was localized on IncHI2-type plasmids, and the *bla*_NDM-1_ gene was localized on IncX3-type plasmids in CR-1025 and CR-1050 and on an IncHI2-type plasmid in CR-1051. Multilocus sequence typing confirmed all strains as sequence type 97 (ST97), and Venn diagram analysis showed close genetic relatedness among the strains.

**Conclusions:**

In conclusion, CR-*E. hormaechei* co-harboring *bla*_NDM-1_ and *mcr-9* exhibits genetic diversity and plasmid mobility, posing a risk of cross-species transmission and clonal spread mediated by mobile genetic elements. Urgent measures are required to curb the dissemination of such multidrug-resistant strains in clinical settings.

## Introduction

The *Enterobacter cloacae complex* (ECC) is an important component of Gram-negative *Enterobacteriaceae*, comprising six subspecies, namely, *Enterobacter asburiae*, *Enterobacter hormaechei*, *Enterobacter kobei*, *Enterobacter ludwigii*, *Enterobacter nimipressuralis*, and *Enterobacter cloacae* (Sanders et al., 1997; Mezzatesta et al., 2012). As an opportunistic pathogen, ECC has been associated with various clinical infections, such as bacteremia, respiratory tract infections, wound infections, and urinary tract infections (Huang et al., 2021).

Carbapenem-resistant *Enterobacteriaceae* (CRE) bacteria pose a serious threat to global public health (Ma et al., 2023), and production of carbapenemases is the main mechanism in CRE clinical isolates (Peng et al., 2022). New Delhi Metallo-β-lactamase (NDM) is a type of Metallo-β-lactamase (MBL) able to hydrolyze most β-lactams (including carbapenems), *bla*_NDM-1_ is often carried in carbapenem-resistant *Enterobacter cloacae complex* (CR-ECC) (Cai et al., 2024; Hu et al., 2022). The emergence of CR-ECC-carrying *bla*_NDM-1_ greatly limits the selection of clinical antibiotics and increases the difficulty of clinical anti-infective treatment. Colistin was once regarded as the last-resort antibiotic for treatment caused by CR-ECC (Halder et al., 2025). However, unfortunately, the activity and efficacy of colistin have been challenged by the global spread of plasmid-mediated colistin resistance genes (*mcr*) (Wu et al., 2023). Currently 10 *mcr* variants (*mcr-1* to *mcr-10*) have been discovered, among them, *mcr-1* and *mcr-9* are the most widespread (Liu et al., 2022; Hussein et al., 2021). Since *mcr-9* was first identified from *Salmonella enterica* in the United States (Carroll et al., 2019), it has been successively detected in 40 countries on six continents (Li et al., 2020), and has become a widely reported variant among CR-ECC (Wu et al., 2023).

This study isolated three ST97 strains of carbapenem-resistant *Enterobacter hormaechei* (CR-*E. hormaechei*) carrying both *bla*_NDM-1_ and *mcr-9* from burned patients, and was conducted to explore the molecular characteristics, antimicrobial resistance (AMR) gene profile and potential transmission mechanism. Furthermore, the genetic background of plasmids was investigated by whole-genome sequencing (WGS).

## Materials and methods

### Sample collection

CRE is defined as resistance to any carbapenem drug, we totally collected 110 CRE from the clinical patients in Zhejiang province, China. The presence of carbapenemase genes and *mcr* genes was screened by PCR amplification using primers described previously (**Supplementary material Table S1**), and the positive products were sequenced using Sanger sequencing. Three strains were detected to carry both *bla*_NDM-1_ and *mcr-9* simultaneously, and they were named as CR-1025, CR-1050, and CR-1051 respectively. Preliminary species identification was achieved by MALDI-TOF MS (Bruker Daltonik GmbH, Bremen, Germany) and 16s rRNA sequencing, all three strains were identified as ECC. And the identification of *E. hormaechei* species was confirmed by WGS.

### Antimicrobial susceptibility testing

The minimum inhibitory concentration (MIC) values of strains against common antibiotics were determined according to the broth dilution method. In accordance with Clinical and Laboratory Standards Institute (CLSI) guidelines, the *Escherichia coli* (*E. coli*) ATCC 25922 was the quality control strain used for the MIC measurement. The results interpreted according to CLSI instructions, while tigecycline resistance was defined according to EUCAST clinical breakpoints.

### Conjugation experiment

The conjugation experiment was carried out using a membrane filter mating experiment as previously described (Zeng et al., 2021; Wang et al., 2015). *E. hormaechei* as the donor strain and *EC600* as recipient strain were mixed on Luria-Bertani agar at a ratio of 1:3, and the mixtures were incubated for 24 h at 35 °C. Transconjugants were selected on MHA plates supplemented with rifampicin (600 µg/ml) and meropenem (1 µg/ml). Colonies that grew on the selective medium were identified by the MALDI-TOF MS and 16S rRNA sequencing. Strains that harbored *mcr-9* and exhibited higher MICs of resistance to carbapenems than *EC600* were defined as the transconjugants. The carrier status of other drug resistance genes and MIC value of the transconjugants were determined by PCR and antimicrobial susceptibility testing.

### Whole-genome sequencing and analysis

Whole-genome sequencing (WGS) was carried out via a combination of the Illumina NovaSeq (Illumina, USA) and PacBio HiFi (Pacific Biosciences, USA) platforms. Concomitantly, sequence analysis yielded high-quality genome assemblies and fully closed genomes of the isolate as well as all its associated plasmids. This entire process was conducted by Majorbio Co., Ltd. (Shanghai, China). In brief, genomic DNA was isolated using a commercial DNA extraction kit (Qiagen, Hilden, Germany). Sequencing libraries were constructed with Illumina Nextera XT kits and subjected to paired-end 150-base sequencing on an Illumina NovaSeq X Plus platform. Furthermore, a dedicated library for PacBio sequencing was prepared using the Rapid Barcoding Sequencing kit and loaded onto an R9.4 flow cell.

For Illumina sequencing data assembly, SOAPdenovo 2.04 ((https://github.com/aquaskyline/SOAPdenovo2) was employed; whereas PacBio data were assembled using Unicycler v0.4.8 (https://github.com/rrwick/Unicycler/releases), Canu v2.2 (https://github.com/marbl/canu), Flye v2.9.2 (https://github.com/mikolmogorov/Flye), NextDenovo v2.5.2 (https://github.com/Nextomics/NextDenovo) and Hifiasm v0.16.1 (https://github.com/chhylp123/hifiasm) to obtain complete whole-genome and plasmid sequences. Genomic sequence annotation was performed using the Pfam Database 33.1 (http://pfam.xfam.org/). Plasmid replicons were identified via PlasFlow 1.1 (https://github.com/smaegol/PlasFlow) and annotated with the PLSDB Database 202106 (https://ccb-microbe.cs.uni-saarland.de/plsdb/). In silico multilocus sequence typing (MLST) was assigned through the PUBMLST database (https://pubmlst.org/databases/). The ChewBBACA method was utilized to identify the core genome, and cg-MLST results were visualized with PHYLOViZ based on the goeBURST algorithm. Acquired antibiotic resistance genes were detected using ResFinder 4.5.0 (https://bitbucket.org/genomicepidemiology/resfinder/src/master/), while virulence genes were predicted via the VFDB Database 20240301 (http://www.mgc.ac.cn/VFs/main.htm). Nucleotide sequences of plasmids or chromosomes carrying *bla*_NDM-1_ and *mcr-9* were compared with homologous sequences using BLAST, and the results were visualized by BRIG (http://brig.sourceforge.net) or Easyfig v2.2.3 (https://github.com/mjsull/Easyfig).

### Nucleotide sequence accession numbers

All sequencing data generated in this study have been deposited in the NCBI Sequence Read Archive under the BioProject accession number PRJNA 1327493 (Biosample: SAMN51284745), PRJNA 1327495 (Biosample: SAMN51284820), PRJNA 1327550 (Biosample: SAMN51284833).

## Results

### Clinical characteristics

Three strains of carbapenem-resistant *E. hormaechei* (CR-*E. hormaechei*) were isolated from elderly male patients, and all three patients were hospitalized with different degrees of burns, two of which were transferred to ICU for treatment due to serious injuries. Two patients (66.7%) received colistin for anti-infection during the treatment period, and all patients (100%) had a history of invasive surgical procedures and multiple antibiotic exposures. Please refer to **Table 1** for specific details.

**Table 1:** Clinical features of carbapenem-resistant *E. hormaechei*.

Isolate number	CR-1025	CR-1050	CR-1051
Ward	ICU	Department of Burns and Plastic Surgery	ICU
Gender	male	male	male
Age(years)	62	70	60
Specimen	wound	blood	wound
Clinical diagnosis	burn	burn	burn
Outcome	died	improved	improved
Invasive operation	yes	yes	yes
Usage of colistin	yes	no	yes
Usage of other antibiotics	PTZ MFX CFS CAZ–AVI AZT	PTZ CFS TGC AMK	PTZ CFS TGC MEM

PTZ, Piperacillin-Tazobactam; MFX, Moxifloxacin; CFS, Cefoperazone-Sulbactam; CAZ-AVI, Ceftazidime-Avibactam; AZT, Aztreonam; TGC, Tigecycline; AMK, Amikacin; MEM, Meropenem.

### Antibiotic resistance phenotype

MICs of 19 antimicrobial agents are shown in **Table 2**. In addition to being resistant to all three carbapenems, CR-1025, CR-1050, and CR-1051 also exhibited resistance to tigecycline, tetracycline, ciprofloxacin, levofloxacin, cefoperazone-sulbactam, piperacillin-tazobactam, cefepime, ceftriaxone, ceftazidime, aztreonam, while remaining but still susceptible to chloramphenicol and amikacin. In contrast, CR-1050 exhibited intermediate susceptibility to both colistin and polymyxin B, whereas CR-1025 and CR-1051 displayed intermediate susceptibility to colistin and full resistance to polymyxin B. All experimental strains have a broad drug resistance spectrum and were classified as multidrug-resistant bacteria.

**Table 2:** Antibiotic susceptibilities of *E. hormaechei* isolates and their transconjugants (μg/mL).

Isolate number	MIC (µg/mL)																		
	CLS	PB	IMP	MEM	ETP	TGC	TC	CAP	CIP	LEV	AMK	GEM	TOB	CFS	PTZ	EFP	CRO	CAZ	AZT
Carbapenem-resistant *E. hormaechei* strains																			
CR-1025	≤1(I)	4(R)	>8(R)	>8(R)	>2(R)	2(R)	>8(R)	≤4(S)	>4(R)	>8(R)	≤8(S)	4(S)	4(S)	>32/8(R)	>64/4(R)	>16(R)	>32(R)	>32(R)	>32(R)
CR-1050	≤1(I)	2(I)	>8(R)	>8(R)	>2(R)	2(R)	>8(R)	≤4(S)	>4(R)	>8(R)	≤8(S)	4(S)	4(S)	>32/8(R)	>64/4(R)	>16(R)	>32(R)	>32(R)	>32(R)
CR-1051	≤1(I)	4(R)	>8(R)	>8(R)	>2(R)	2(R)	>8(R)	≤4(S)	>4(R)	>8(R)	≤8(S)	8(I)	8(I)	>32/8(R)	>64/4(R)	>16(R)	>32(R)	>32(R)	>32(R)
*E. hormaechei* transconjugant strains																			
*EC600*	<0.5(I)	<0.5(I)	<0.5(S)	0.5(S)	<0.5(S)	<0.5(S)	≤1(S)	≤2(S)	<0.5(S)	<0.5(S)	≤2(S)	≤1(S)	≤1(S)	≤8/4(S)	≤8/4(S)	<0.5(S)	<0.5(S)	<0.5(S)	≤1(S)
J1025	≤1(I)	1(I)	>8(R)	>8(R)	>2(R)	2(R)	>8(R)	≤4(S)	>4(R)	>8(R)	≤8(S)	≤2(S)	≤2(S)	>32/8(R)	>64/4(R)	>16(R)	>32(R)	>32(R)	>32(R)
J1050	≤1(I)	1(I)	>8(R)	>8(R)	>2(R)	≤1(S)	≤2(S)	≤4(S)	≤0.5(S)	≤1(S)	≤8(S)	≤2(S)	≤2(S)	>32/8(R)	>64/4(R)	>16(R)	>32(R)	>32(R)	>32(R)
J1051	≤1(I)	1(I)	8(R)	>8(R)	>2(R)	≤1(S)	>8(R)	≤4(S)	≤0.5(S)	≤1(S)	≤8(S)	4(S)	8(I)	>32/8(R)	>64/4(R)	>16(R)	>32(R)	>32(R)	>32(R)

CLS, Colistin; PB, Polymyxin; IMP, Imipenem; MEM, Meropenem; ETP, Ertapenem; TGC, Tigecycline; TC, Tetracycline; CAP, Chloramphenicol; CIP, Ciprofloxacin; LEV, Levofloxacin; AMK, Amikacin; GEM, Gentamicin; TOB, Tobramycin; CFS, Cefoperazone-Sulbactam; PTZ, Piperacillin-Tazobactam; EFP, Cefepime; CRO, Ceftriaxone; CAZ, Ceftazidime; AZT, Aztreonam.

### Results of plasmid conjugation test

The conjugation success rate was 100% (3/3). Both *bla*_NDM-1_ and *mcr-9* were able to transfer from donor to recipient, confirming the horizontal transmission of the plasmid. After conjugation, the transconjugants showed a significant increase in resistance to carbapenems, while the MIC values for the other drugs exhibited varying degrees of changes. The specific results are detailed in **Table 2**.

### Results of WGS

#### Resistome and virulence genes in isolates and location

In addition to co-harbored *mcr-9* and *bla*_NDM-1_ genes, CR-1025 and CR-1050 also carried 12 other antimicrobial resistance genes mediating resistance to aminoglycosides (*aadA1*, *ant(2’’)-Ia*, *aadA2b*, *aadA3*), β-lactams (*bla*_ACT-5_, *bla*_CTX-M-9_, *bla*_SHV-12_), fosfomycin (*fosA*), quinolones (*qnrA1*), sulphonamides (*sul1*), tetracycline (*tet(A)*) and trimethoprim (*dfrA16*). By contrast, CR-1051 harbored 9 other resistance genes. These resistance genes were distributed on different genetic carriers of bacteria (one chromosome and multiple plasmids). Some plasmids even carry multidrug-resistant genes, while certain drug-resistant genes were present on multiple plasmids.

**Figure 1:**
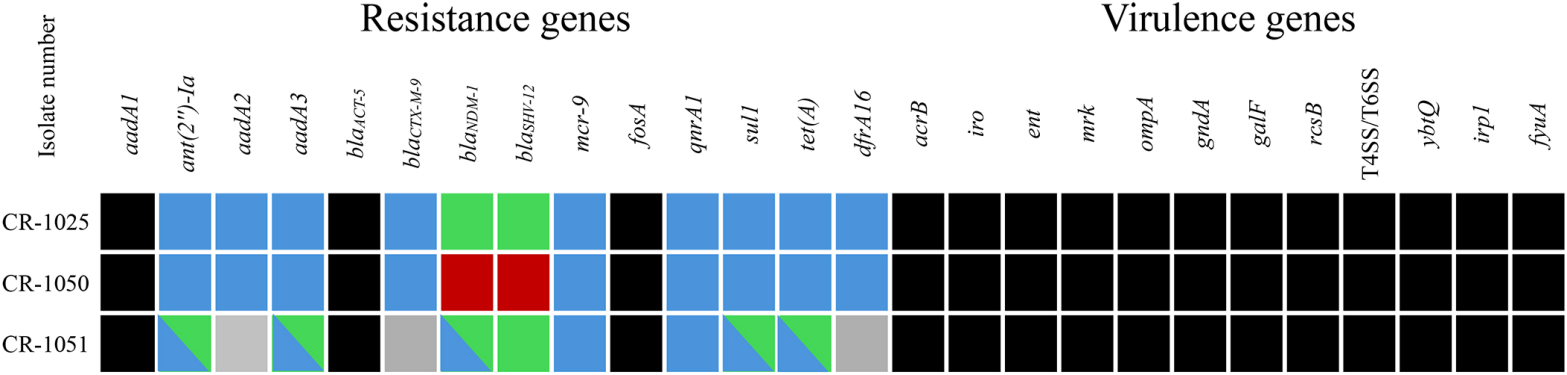
Profile of resistance and virulence genes in CR- *E. hormaechei*. Black: Present and located on chromosome; Blue: Present and locate on plasmid A; Red: Present and locate on plasmid B; Green: Present and locate on plasmid C; Grey: Absent; Two colors: both of the two different plasmids corresponding to the different colors carry this gene. T4SS/T6SS: Type IV/VI secretion system.

In addition to resistance genes, CR-1025, CR-1050 and CR-1051 also carried many virulence genes on the chromosome. The profiles of their virulence genes are complex and are associated with such as bacterial adhesion, colonization, transcriptional regulation, and other biological processes. Please refer to **Figure 1** for specific details.

#### Genetic characteristics of the plasmid-borne *mcr-9*

**Figure 2:**
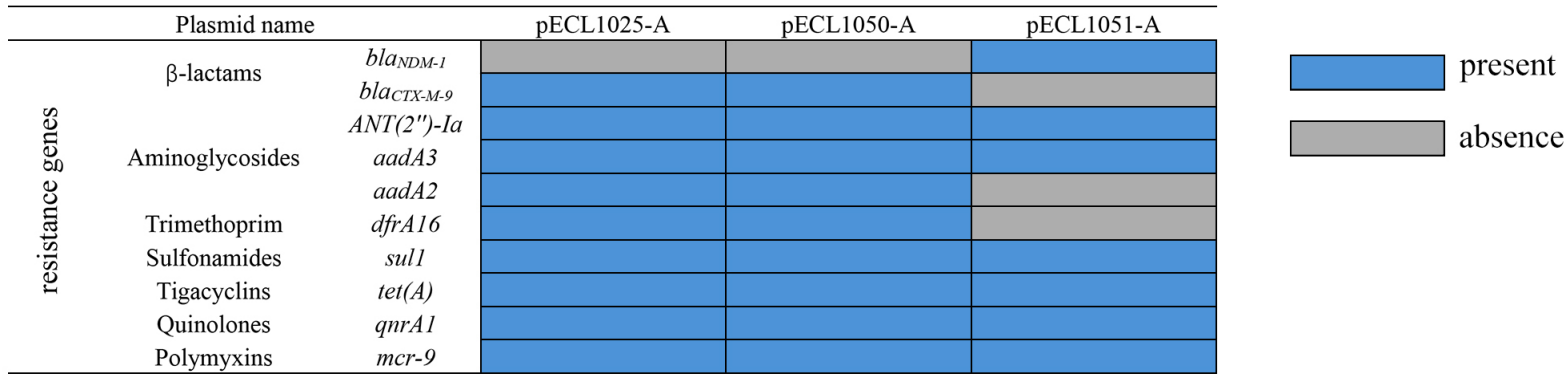
Distribution of antimicrobial resistance genes in plasmids carrying *mcr-9*. Blue: Resistance gene present in the corresponding *mcr-9*-harboring plasmid; Gray: Resistance gene absent in the corresponding *mcr-9*-harboring plasmid.

**Figure 3:**
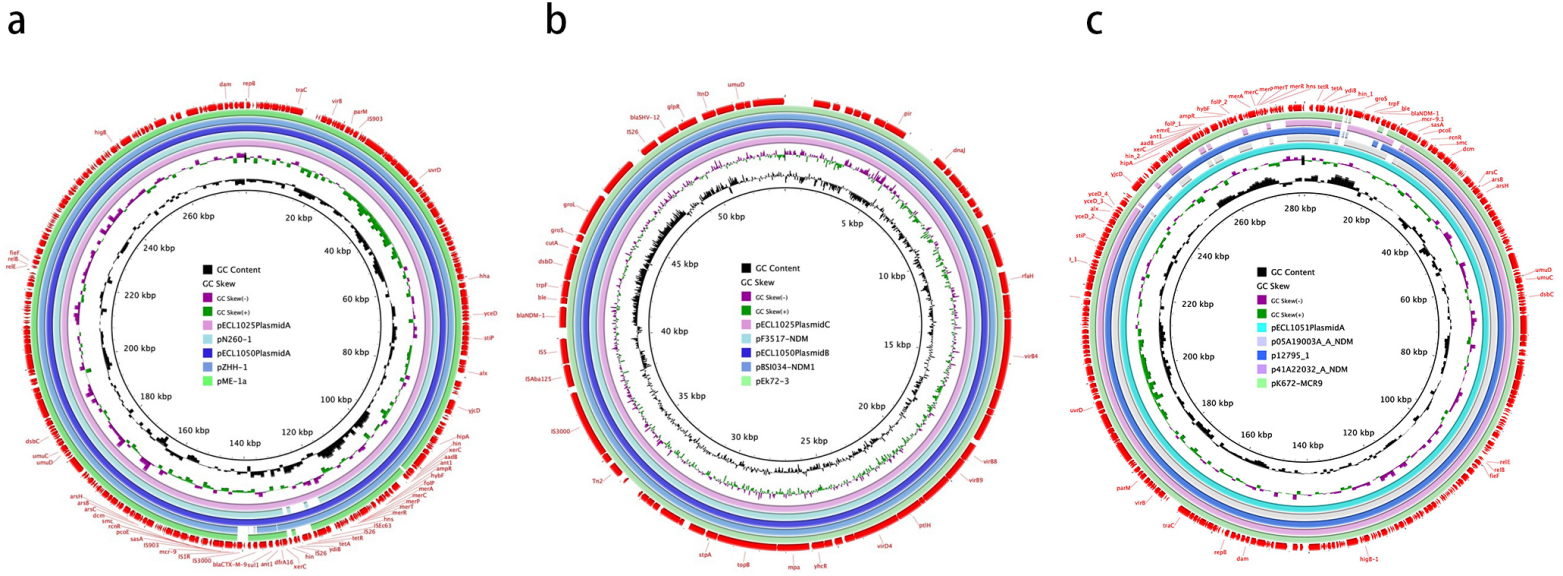
Schematic maps of plasmids harboring *bla*_NDM-1_ and (or) *mcr-9*. **(a)**. Map of the plasmids pECL1025-A (this study), pECL1050-A (this study), pN260-1 (accession no. AP023448.1), pzHH-1 (accession no. CP059712.1), and pME-1a (accession no. CP041734.1). **(b)**. Map of the plasmids pECL1025-C (this study), pECL1050-B (this study), pF3517-NDM (accession no. CP137176.1), pBSI034-NDM1 (accession no. MN937240.1), and pEk72-3 (accession no. CP088232.1). **(c)**. Map of the plasmids pECL1051-A (this study), p05A19003A_A_NDM (accession no. PV023031.1), p12795_1 (accession no. CP083854.1), p41A22032_A_NDM (accession no. PV023161.1), and pK672-MCR9 (accession no. CP183850.1).

Whole-genome sequencing revealed that isolates CR-1025, CR-1050, and CR-1051 harbored 4, 3, and 4 plasmids, respectively. Plasmids in each isolate were designated alphabetically (A to D) in descending order of size; thus, CR-1025 and CR-1051 contained plasmids A–D, while CR-1050 contained plasmids A–C. In all three isolates, the largest plasmid (designated A) carried the *mcr-9* gene and corresponded to pECL1025-A, pECL1050-A, and pECL1051-A, respectively. These *mcr-9*-positive plasmids belonged to the IncHI2 replicon type and carried genes involved in replication (*repB*), stability (*parM*), and conjugative transfer (*tra* and *virB*). CR-1025 and CR-1050 shared two pairs of homologous plasmids: one pair (pECL1025-A and pECL1050-A) carried *mcr-9* and was 278,515 bp in size with a GC content of 46.30%; the other pair (pECL1025-C and pECL1050-B) carried *NDM-1* and measured 54,035 bp with a GC content of 49.03%. Notably, pECL1051-A—also an IncHI2 plasmid—harbored *mcr-9* along with six additional resistance genes, including *NDM-1*. Key characteristics of these plasmids are summarized in **Table 3**; **Figure 2**, and schematic maps are provided in **Figure 3**.

**Table 3:** Characteristics of plasmid carrying the *mcr-9* gene and (or) *bla*_NDM-1_.

Isolate number	Plasmid numbers	Plasmid name	Colistin resistance gene	Carbapenem resistance genes	Plasmid size (bp)	Plasmid replicon type	G+C content (%)
CR-1025	4	pECL1025-A	*mcr-9*	–	278,515	IncHI2	46.3
		pECL1025-C	–	*bla*_NDM-1_	54,035	IncX3	49.03
CR-1050	3	pECL1050-A	*mcr-9*	–	278,515	IncHI2	46.3
		pECL1050-B	–	*bla*_NDM-1_	54,035	IncX3	49.03
CR-1051	4	pECL1051-A	*mcr-9*	*bla*_NDM-1_	281,800	IncHI2	46.66

#### Analysis of the genetic environment of *mcr-9*

**Figure 4:**
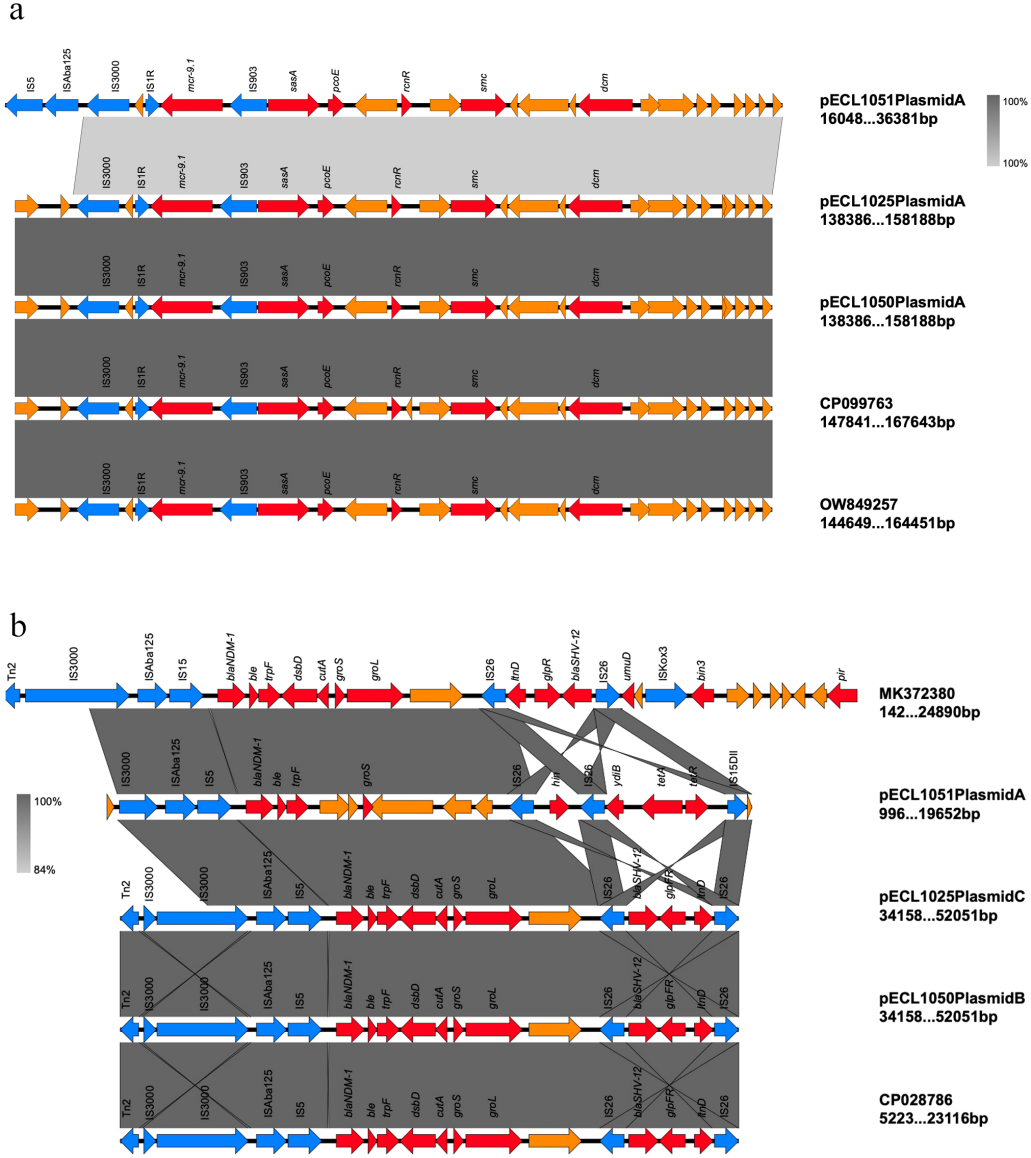
Linearized analyses for genetic environment of *mcr-9* and *bla*_NDM-1_. **(a)**. *mcr-9* harbored by pECL1051-A, pECL1025-A, and pECL1050-A in this study. **(b)**. *bla*_NDM-1_ harbored by pECL1051-A, pECL1025-C, and pECL1050-B in this study.

The genetic environments of *mcr-9* in this study were highly similar and *mcr-9* was located in an ~4-kb region surrounded by two insertion sequences *IS903* and *IS1R*, there was also an *IS3000* downstream. The region upstream of *mcr-9* included the conserved gene structure, *rcnR*- *pcoE*- *sasA*, but lacked the downstream structure *wbuC*. These genes may be involved in the regulation of plasmid-related functions, such as metal ion transport regulation and plasmid replication regulation, and might influence the expression and function of *mcr-9* to some extent, also reflecting, also reflecting the cooperative and interactive relationships between genes on the plasmid. These elements may play a crucial role in transferring *mcr-9* (**Figure 4a**). A similar structure has been found in some IncHI2 plasmids such as p123 (Accession no. CP099763) and p1 (Accession no. OW849257).

#### Analysis of the plasmids and genetic environment of *bla*_NDM-1_

The genetic structures of pECL1025-C and pECL1050-B were highly homologous, and both belong to the IncX3 type. The *bla*_NDM-1_ gene, accompanied by *ble*_MBL_ (bleomycin resistance gene), was located downstream of an *ISAba125* element. This *ISAba125* was disrupted by the insertion of *IS5*; furthermore, an *IS3000* and a truncated *Tn2* element were found upstream of the element. They also harbored *bla*_SHV-12_, which was flanked by two *IS26* elements. However, pECL1051-A—an IncHI2 plasmid approximately five times the size of pECL1050-B—does not carry *bla*_SHV-12_. Its downstream structure varied greatly, and there was no *Tn2* upstream. For specific details, refer to **Figure 4b**.

#### Homology analysis comparison

**Figure 5:**
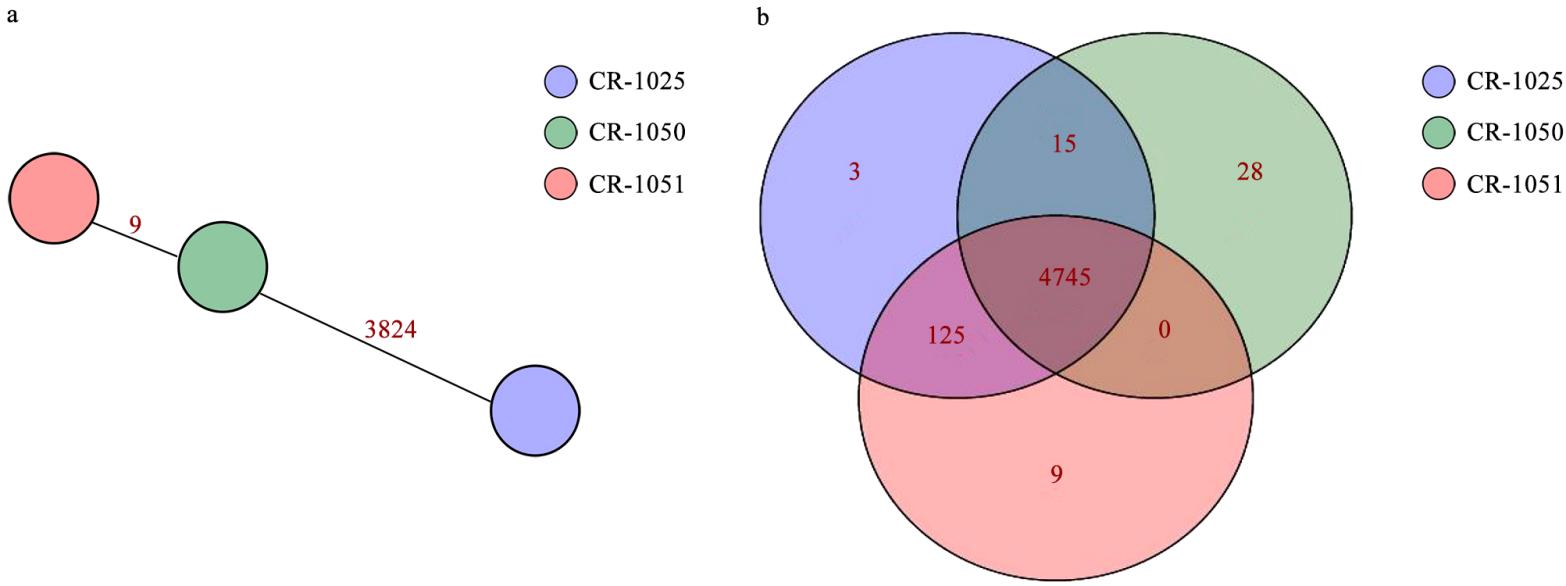
Results of cg-MLST and homologous gene analysis. **(a)**. Minimum spanning tree based on the core-genome MLST. The allelic distance between strains was represented by numbers. **(b)**. Homologous gene analysis results (Venn). Each colored ellipse in the figure represents a strain; the numbers in the intersecting parts among strains indicate the number of common gene families, and the numbers in the individual parts of each strain indicate the number of unique gene families of that strain.

MLST analysis identified all strains as sequence type ST97. Core-genome MLST (cg-MLST) further resolved their genetic relatedness (**Figure 5a**), revealing 9 and 3,824 allelic differences between CR-1050/CR-1051 and CR-1050/CR-1025, respectively. Based on homologous gene clusters (**Figure 5b**), the three strains shared 4,745 core gene families, while harboring 3 (CR-1025), 28 (CR-1050), and 9 (CR-1051) unique families. These results demonstrate a high overall genetic similarity among the *E. hormaechei* strains, with CR-1050 and CR-1051 being most closely related.

## Discussion

*bla_NDM-1_* and *mcr-9* have been detected in various bacteria worldwide (Hem et al., 2024; Li et al., 2023; Cahill et al., 2023). However, few reports have performed a detailed genomic analysis of CR-*E. hormaechei* with *bla*_NDM-1_ and *mcr-9*, simultaneously. In this study, three CR-*E. hormaechei* strains carrying both *bla*_NDM-1_ and *mcr-9* were isolated from three patients with varying degrees of burns.

*mcr-9* has been identified in several CRE backgrounds since it was described in 2019 (Macesic et al., 2021), and its prevalence in clinical isolates from Asia and Europe has increased annually suggesting the potential for a global outbreak. *mcr-9* is located on IncHI2 plasmids (Chen et al., 2022), consistent with the findings of this study. pECL-1025 plasmid A, pECL-1050 plasmid A and pECL-1051 plasmid A share a similar *mcr-9* gene background, the conserved region encodes nickel-cobalt efflux transporters (*rcnR*) and sensory protein kinases (*pcoE*). Sequence alignment showed that there was a similar structure to the reference plasmid p123 (Accession no. CP099763) from a *Salmonella enterica* in 2023 from Belgium and plasmid p1 (Accession no. OW849257) from a *Citrobacter freundii* in 2022 from Spain. This indicates that the abundance of mobile genetic elements endows the gene structure in the *mcr-9* region with high dynamism and plasticity, providing favorable mobility and dissemination conditions for the *mcr-9* gene. Similarly, the *mcr-9*-like structure is not only identified in the *Enterobacter cloacae complex* (ECC) but also detected in other species such as *Salmonella* and *Citrobacter*. This observation further highlights the high risk of *mcr-9* gene transfer among different plasmids or across the genomes of distinct bacterial species. This underscores the urgent need for close monitoring to determine the detection rate of *mcr-9* and for effective action to control its further spread.

The *bla*_NDM-1_ gene is commonly found on high-frequency transfer plasmids such as IncX3 and IncC, the *IS5* transposon has made a significant contribution to its dissemination (Uz Zaman et al., 2018), and a 100% conjugation success rate further validates this conclusion. The emergence of *bla*_NDM-1_ is one of the primary drivers of carbapenem-resistant *Enterobacteriaceae* (CR-ECC). The co-expression of aminoglycoside resistance, trimethoprim resistance, sulfonamide resistance, quinolone resistance, and β-lactamase genes confers a multidrug-resistant (MDR) phenotype on the strain, exerting pressure on clinical antibiotic selection. Interestingly, in the present study, *bla*_CTX-M-9_ and *mcr-9*, as well as *bla*_SHV-12_ and *bla*_NDM-1_, exhibit a strong association respectively. *bla*_CTX-M-9_ has previously been identified as the most prevalent extended-spectrum β-lactamase (ESBL) gene among hospital-acquired CRE in China (Zhou et al., 2018). *bla*_SHV-12_ has also been frequently detected in the *Enterobacter cloacae complex* (ECC) (Houkes et al., 2025). This indicates that genetic elements can form “antibiotic resistance gene clusters” through homologous recombination or co-integration events. The simultaneous transmission of such clusters across strains and species would constitute a significant public health threat.

Many *Enterobacter hormaechei* strains are characterized by high antimicrobial resistance but low virulence (Davin-Regli et al., 2019). In this study, the virulence genes carried by *E. hormaechei* include *acrB*, T4SS/T6SS, and *rcsb*. These genes are associated with bacterial colonization and efflux systems, enabling the bacteria to develop resistance to cytotoxic substances and thereby reduce the impact of antibiotics on them. Although these virulence factors are located on chromosomes and have limited transmission rates compared to mobile elements such as plasmids, their synergistic effects on drug resistance could potentially enable *E. hormaechei* to possess both high drug resistance and high virulence simultaneously. The emergence of such strains would significantly increase treatment challenges. Therefore, the potential pathogenicity risk of ECC also warrants attention.

This study reports the first detection of CR-ECC with ST97 suggests that this type is likely the predominant clonal group in this region. Furthermore, all three patients received treatment in the burn unit, indicating a probable nosocomial outbreak within that department. However, this requires further investigation incorporating temporal and epidemiological data.

## Conclusions

In summary, we first report the genomic characteristics of three ST97 clinical *E. hormaechei* strains carrying both *bla_NDM-1_* and *mcr-9*. The CR-*E. hormaechei* strains have a broad drug resistance spectrum, posing a challenge to clinical treatment. Whether it is *bla*_NDM-1_ or *mcr-9*, if no measures are taken to control them, they will be spread widely and significantly, in a matter of time. We should conduct routine genomic surveillance to effectively curb the spread of drug-resistant bacteria in the region.

## Data Availability

The datasets presented in this study can be found in online repositories. The names of the repository/repositories and accession number(s) can be found in the article/**Supplementary Material**.
