# Supplementary material for: Co-occurrence of mcr-9 and blaNDM-1 in carbapenem-resistant Enterobacter hormaechei from burn patients
Source: Front Cell Infect Microbiol. 2026 Feb 13;16:1742596. doi: 10.3389/fcimb.2026.1742596 (PMC12946085; doi:10.3389/fcimb.2026.1742596)
Supplement: Supplementary file 1 [file Table1.docx]

**Supplementary material**

**Table 1 Primer used in this study.**

| Primer name | *Primer-F*  *Primer-R* | Primer sequence | |
| --- | --- | --- | --- |
| *NDM* | *NDM-F* | TCGCCCCATATTTTTGCTACAG |  |
|  | *NDM-R* | CGATCCTTCCAACTCGTCGC |  |
| *IMP-4* | *IMP-4-F* | ACCGCAGCAGAGTCTTTGCC |  |
|  | *IMP-4-R* | ACAACCAGTTTTGCCTTACC |  |
| *IMP-8* | *IMP-8-F* | GTTTTATGTGTATGCTTCC |  |
|  | *IMP-8-R* | AGCCTGTTCCCATGTAC |  |
| *KPC* | *KPC-F* | TGTCACTGTATCGCCGTC |  |
|  | *KPC-R* | CTCAGTGCTCTACAGAAAACC |  |
| *VIM-1* | *VIM-1-F* | AGTGGTGAGTATCCGACAG |  |
|  | *VIM-1-R* | ATGAAAGTGCGTGGAGAC |  |
| *VIM-2* | *VIM-2-F* | ATGTTCAAACTTTTGAGTAAG |  |
|  | *VIM-2-R* | CTACTCAACGACTGAGCG |  |
| *OXA-48* | *OXA-48-F* | TTGGTGGCATCGATTATCGG |  |
|  | *OXA-48-R* | GAGCACTTCTTTTGTGATGGC |  |
| *mcr-9* | *mcr -9-F*  *mcr -9-R* | CTTTCCATAACAGCGAGACAC  GTATCCTTCCTGCCATCCTC |  |
|  |  |  |  |
